# Validation of the Measure of Case‐Discussion Complexity and the Metric for the Observation of Decision‐Making for Streamlining Workflow and Evaluating Decision‐Making in US Tumor Boards

**DOI:** 10.1002/cam4.71970

**Published:** 2026-05-29

**Authors:** T. Soukup, N. Lajmi, M. S. Prime, B. W. Lamb, W. Zhang, G. Markollari, R. Ortiz, S. Perrigo, K. Stuart, M. Wallace, K. Noyes, R. D. Hammer

**Affiliations:** ^1^ Department of Surgery and Cancer, Faculty of Medicine Imperial College London London UK; ^2^ Clinical Value Design & Validation Roche Information Solutions Santa Clara California USA; ^3^ Clinical Decision Support and Algorithms Roche Information Solutions, Roche Diagnostics International Rotkreuz Switzerland; ^4^ Department of Urology Barts Health NHS Trust London UK; ^5^ Barts Cancer Institute Queen Mary University of London London UK; ^6^ Department of Urology University College London Hospitals NHS Foundation Trust London UK; ^7^ Department of Pathology and Anatomical Sciences University of Missouri Columbia Missouri USA; ^8^ Department of Surgery, Jacobs School of Medicine and Biomedical Sciences University at Buffalo Buffalo New York USA

## Abstract

**Background:**

Clinical guidelines for cancer care recognize multidisciplinary tumor boards (MTBs) as a gold standard for providing quality of care for cancer patients. However, the requirements and workflow for MTBs varies across countries, influencing their roles in decision making, care efficiency, and medical education. This study compares validity and performance of MDT evaluation tools, Measure of case‐Discussion Complexity (MeDiC) and Metric for the Observation of Decision‐Making (MODe) in the UK versus US.

**Methods:**

MeDiC, developed to assess case complexity, and MODe, designed to evaluate decision‐making quality, were applied to 555 cases and 104 MTB meetings from urological and gynecological tumor boards at an academic cancer center between December 2021 and June 2024. MeDiC was used to assess clinical complexity through pathology, patient, and treatment factors, while MODe evaluated decision‐making quality based on patient information and team contributions. All assessors underwent formal training. Data analysis included reliability testing using intraclass correlation coefficients (ICCs), Kappa, Cronbach's alpha, and bootstrapping was used to estimate confidence intervals for correlation estimates.

**Results:**

MeDiC demonstrated good reliability (ICC = 0.849) in the US MDT and identified key factors contributing to complexity, such as malignancy and significant comorbidities. The tool also revealed that case discussion duration was not significantly associated with complexity levels. MODe similarly showed moderate reliability (ICC = 0.628), with high correlations between decision‐making quality and contributions from core specialties such as medical oncologists.

**Conclusions:**

The validity of MeDiC and MODe in the US was similar to the tools' performance in the UK settings. However, case complexity metrics demonstrated significant selection of more complex cases for US MDT presentation compared to UK MDTs.

## 1 Introduction

Globally, multidisciplinary tumor boards (MTBs) have become a critical component of cancer care, providing a platform where specialists collaborate to address the increasingly complex needs of cancer patients. MTBs allow teams of healthcare professionals—including medical and surgical oncologists, radiologists, pathologists, genetic counselors, nurses, and ancillary services (e.g., social workers, palliative care specialists)—to review key aspects of a patient's case (such as tumor characteristics, medical history, imaging, comorbidities, and psychosocial issues) in order to formulate comprehensive treatment plans. This multidisciplinary approach is widely recognized for optimizing care, improving adherence to treatment guidelines, and enhancing patient outcomes [1, 2, 3, 4, 5].

However, the growing global cancer burden, coupled with an aging population and the rapid increase in available treatment options, has resulted in a significant increase in the complexity of decision‐making for cancer providers [6, 7]. At the same time, mounting financial pressures on healthcare systems limit time for MTB discussions and physician‐to‐physician communication [8, 9]. Moreover, preparing for MTB meetings remains resource‐intensive and laborious, demanding substantial time and effort from the care team, particularly radiologists and pathologists, which further limits their participation in MTBs; even as digital solutions are emerging to help streamline workflows, resource limitations continue to be a critical barrier [10, 11].

To address these challenges, prioritizing complex cancer cases has emerged as a key strategy to ensure that MTBs maintain high standards of care within a limited allocated time, particularly in countries where tumor board discussions were previously mandated for all cancer cases [12]. In the United States (USA), unlike in the United Kingdom (UK), for example, tumor boards are not required to discuss all cancer cases with MDT preparation often led by nurse coordinators, and meetings also serving an educational role for residents to illustrate rare or unusual cases [12, 13].

This study focuses on the validation of two complementary tools for evaluating MDT function: the Measure of Case‐Discussion Complexity (MeDiC) [14, 15] and the Metric for the Observation of Decision‐Making (MODe) [16, 17] for use during the US MTBs. MeDiC was first developed for UK tumor boards in 2020 [14] and later validated in Sweden [15]. Previous studies established MeDiC as an evidence‐based, expert‐developed tool that reliably assesses cancer case complexity while enhancing treatment planning efficiency. Its initial application demonstrated the tool's feasibility in real‐world clinical settings. Here, we extend its validation to a US cancer care setting, where members are not reimbursed for MTB participation, only selected cases are discussed, and access to care varies across different care facilities.

We also extend the validation of the MODe tool to the US population. Developed in 2011 for UK tumor boards [16] and later applied in Europe and Australia [17], MODe has consistently demonstrated reliability in evaluating decision‐making quality in MTBs. While MeDiC focuses on assessing case complexity, MODe evaluates the quality of decision‐making by analyzing key factors such as the comprehensiveness of patient information—case history, radiology, pathology—and contributions from core disciplinary groups within the tumor board. MODe also functions as a self‐assessment tool for team audit and feedback, facilitating continuous improvement in decision‐making processes [18]. By capturing key aspects of decision‐making dynamics, MODe provides insights into how factors such as meeting preparation, case prioritization, team training, and improved chairing influence decision‐making quality, as assessed through pre/post evaluations.

To our knowledge, this is the first validation of these tools in the US healthcare system, where tumor boards are not uniformly mandated and where the integration of clinical complexity can be more fragmented. Such US‐based validation is critical for determining the tools' applicability in a setting with different institutional and systemic pressures, providing new insights into how MTBs can optimize decision‐making in the face of increasing case complexity.

## 2 Methods

### 2.1 Study Design and Setting

Data for this longitudinal observational study was collected at an academic medical center in Columbia, Missouri between December 2021 and June 2024. Two tumor boards took part: urological and gynecological, including all MTB members who regularly attend the meetings which were held in person. The research was approved by the local institutional review boards prior to data collection.

### 2.2 Ethics Approval and Consent to Participate

This study was reviewed and approved by the University of Missouri Institutional Review Board (IRB). All participants provided written informed consent prior to their participation.

### 2.3 Availability of Data and Materials

The copyright of the MeDiC tool and items rests with Soukup, Sevdalis, and Green, and the final version of the tool with item description can be obtained from Soukup on request.

### 2.4 Patient and Public Involvement

Patient and public were not involved in this study.

### 2.5 Description of the Tools

#### 2.5.1 Measure of Case‐Discussion Complexity

Measure of case‐Discussion Complexity (MeDiC) [14] was developed through a multiphase research process over 18 months with input from cancer specialists throughout and at a national level in the UK. It demonstrated evidence of reliability and validity in its scores, as well as feasibility in utilization by both medically and non‐medically trained assessors. MeDiC captures clinical complexity, including pathology, patient‐specific factors and treatment for each patient discussed in the tumor board. It uses a checklist‐based scoring system with weighted items, ensuring a granular assessment of complexity. The tool consists of 26 items, with scores ranging from a minimum of 0 to a maximum of 54. It can be applied observationally (in person or from recorded discussion) or directly from clinical records, allowing flexibility depending on the aim: for care prioritization, it should be conducted prior to the meeting, whereas for research purposes, it can be conducted in either manner.

#### 2.5.2 Metric for Observation of Decision‐Making

Metric for Observation of Decision‐making (MODe) [16] is used to assess decision‐making process by multidisciplinary teams. It has been applied previously to assess different tumor boards and has demonstrated good validity and reliability [16, 17]. It captures the following aspects observationally:

1. Quality of presented patient information, which includes 6 variables scored on a behaviorally anchored 5‐point scale (patients' case history, radiology, pathology, psychosocial issues, co‐morbidities, views on treatment options). Additionally, the MODe evaluated whether the patient has been seen by a team member prior to the meeting, recorded as a binary variable (Y/N). The sum of the scores for all 7 variables represents overall quality of presented information (ranged from 7 to 32), with higher scores indicating better quality.
2. Quality of contribution to case‐reviews, which originally includes 5 variables scored on a behaviorally anchored 5‐point scale, representing contributions made by surgical oncologists, medical oncologists, radiologists, pathologists, and cancer nurse specialists. To align the tool with the core professional groups in the participating tumor boards in the US, we have included an additional 3 variables representing the contribution from clinical trials specialists (considering only site‐specific trials, not all available trials), radiation oncologists, and geneticists. The sum of the scores for all 8 variables represents overall quality of contribution (ranging from 8 to 37) for a patient, with the higher scores indicating better quality.
3. Team ability to reach a treatment recommendation, as a dichotomous variable.

### 2.6 Assessors and Training

Training in the use of the two tools was undertaken by all assessors (*N* = 7) prior to the formal scoring during the study (distribution of assessors is presented in Table S1). Such training is essential for the effective use of the tool, and it is a general principle for instruments assessing human factors in clinical environments [19]. Training for each tool was delivered by our team (TS) and took place over two full days per tool (4 days in total). It included structured teaching, guided practice, and calibration exercises using video recordings of MTBs and constructed case scenarios representing a range of clinical complexity. The training covered: (1) explanation of the domains, scales and their anchors, (2) background reading of peer‐reviewed literature on the tool, and (3) calibration of scoring against an expert evaluator (TS) via scoring a set of MDT videos. Following the training and prior to the data collection for the study, all assessors practiced scoring the tumor boards in real clinical environments for two MTB meetings. Proficiency in scoring was set as an achievement of inter‐assessor reliability of 0.70 or higher between the trainee and expert assessor across the two instruments; this was met. Second assessors rated 20% of case‐discussions (which corresponds to 20% of the meetings) for each tool respectively, and their scores were calibrated against the main assessor in the training (TS), and for the study (SP, GM). Each evaluator was blind to the other evaluators' observations. For MODe, any major inconsistencies on the subset of the data scored for inter‐assessor reliability were resolved through checking for errors and the discussion between the main assessor (SP) and the 2nd assessors (SP, GM).

To reduce bias, we followed the guidance for observational studies involving MTBs published previously [20, 21]. Specifically, the o*bserver bias* was addressed and reliability of evaluations on the two instruments was ensured by having a subset of cases scored by the evaluators who were all trained in the use of the instruments (for MODe: SP was main assessor with KS (attended all MTB in person), WZ RO as second assessors; for MeDiC: GM was main assessor with MW, RO, WZ as second assessors). During data collection, each evaluator was blind to the other evaluators' observations. To reduce the *Hawthorne effect*, i.e., teams changing their usual behavior due to being observed, we adopted a long‐term approach by observing each team for a prolonged period of time, and we excluded the first two meetings in each team from the analysis as they were designed to allow the members to get used to being observed and induce habituation. We also ensured that observations were made discreetly by addressing any factors (i.e., observers could not interact with MTB members), reducing any constant reminders of the observation process. This approach allowed the members to “forget” about being observed and continue their working practices as usual. Due to logistical feasibility, assessor availability, and workload considerations, assessors conducted their evaluations either in person, remotely via videoconference, or asynchronously through Electronic Medical Record (EMR) review.

### 2.7 Data Analysis

#### 2.7.1 Reliability Assessments

Reliability between two assessors for both tools was assessed using Intraclass Correlation Coefficients (ICC) for continuous variables and Kappa statistics for categorical items, with generally accepted reliability thresholds of 0.70 and above. Cronbach's Alpha was calculated to assess the internal consistency of each tool (MeDiC: Tables 1 and 2; MODe: Table 3).

**TABLE 1:** Reliability, convergent validity, and external validity for the MeDiC tool.

	MeDiC tool: Complexity factor	Item weighing	Assessor reliability	Item reliability	Item frequency		Correlation^e^						Soukup et al. item evaluation (UK)
			Kappa	Cronbach's Alpha if item removed^a^	Count	%	Item‐MeDiC total^d^				Case duration		
							*r* (unadjusted)	% variability explained	*r* (adjusted^c^)	% variability explained	*r*	% variability explained	
Pathology factors													
1	Malignancy	1	0.755	0.579	494	89	0.239*	6	0.168*	3	0.100*	1	Excellent
2	Invasive component	1	0.759	0.573	297	54	0.357*	13	0.259*	7	0.047	0	Excellent
3	Multiple cancers (incl. multiple primaries)	1	0.493	0.582	48	9	0.218*	5	0.112*	1	0.082	1	Excellent
4	Increased size (T3, T4)	1	0.675	0.579	263	47	0.241*	6	0.192*	4	0.161*	3	Excellent
5	Nodes affected	1	0.704	0.573	171	31	0.400*	16	0.214*	5	0.118*	1	Excellent
6	Mets (local or distant)	1	0.681	0.565	173	31	0.544*	30	0.312*	10	0.130*	2	Excellent
7	Advanced stage, progressive	1	0.729	0.570	145	26	0.434*	19	0.286*	8	0.135*	2	Excellent
8	Unusual or rare tumor type	4	0.720	0.593	41	7	0.124*	2	0.263*	7	0.140*	2	Excellent
9	Residual tumor	1	0.714	0.584	35	6	0.133*	2	0.077	1	0.003	0	Moderate
10	Recurrence	1	0.758	0.578	66	12	0.317*	10	0.171*	**3**	0.029	0	Moderate
Patient factors													
11	Previous history of cancer	1	0.747	0.574	190	34	0.333*	11	0.220*	5	0.152*	2	Excellent
12	Previous oncological treatment	1	0.612	0.576	128	23	0.334*	11	0.160*	3	−0.052	0	Excellent
13	Significant surgical history	3	0.693	0.558	302	54	0.361*	13	0.540*	29	−0.057	0	Excellent
14	Significant physical comorbidity (incl. poor PS^f^)	3	0.893	0.560	323	58	0.314*	10	0.535*	29	−0.018	0	Excellent
15	Mental health and cognitive comorbidity	4	0.648	0.584	65	12	0.184*	3	0.418*	17	−0.058	0	Moderate
16	Socio‐economic issues	3	0.737	0.583	56	10	0.192*	4	0.278*	8	0.020	0	Moderate
17	Patient choice and family opinion*	1	0.721	0.578	79	14	0.263*	7	0.214*	5	0.024	0	Moderate
18	Lifestyle risks	3	0.927	0.588	75	14	0.161*	3	0.268*	7	−0.027	0	Poor
Treatment factors													
19	Diagnostic uncertainty and inconclusiveness of diagnostic tests	1	0.720	0.586	58	11	0.086*	1	0.046	0	0.008	0	Excellent
20	Unusual anatomy/distribution of tumor	1	0.695	0.583	20	4	0.149*	2	0.084	1	0.100*	1	Excellent
21	Conflict of opinions about treatment options	4	1.000	0.592	9	2	0.010	0	0.051	0	0.009	0	Excellent
22	Further tests and patient assessment needed	1	0.665	0.582	169	31	0.232*	5	0.125*	2	0.062	0	Moderate
23	Treatment toxicity and contraindications	3	0.654	0.583	9	2	0.144*	2	0.141*	2	0.074	1	Moderate
24	Further input needed from other specialties	1	0.621	0.580	106	19	0.235*	6	0.140*	2	−0.016	0	Moderate
25	Pathway does not account for patients specific situation	4	0.793	0.588	13	2	0.074	1	0.141*	2	0.074	1	Poor
26	Trial eligibility	1	0.758	0.586	30	5	0.074	1	−0.030	0	0.022	0	Poor
Total MeDiC score (sum of items 1 to 26)^b^		—	0.849^b^	—	—	—	—	—	—	—	0.126*	2	Excellent

*Note:* *N* = 555 discussions. *N* = 104 meetings. *N* of cases double‐rated = 112 (20% of the total *N*). *N* of meetings double rated = 22 (21.15% of the total N of meetings). Green = good measure of complexity. Yellow = fair. Red = weak and could be removed. Percentage values have been rounded to the nearest integer for ease of reading. MeDiC Copyright 2017 Soukup Sevdalis Green.

* *p* < 0.05.

^a^ Cronbach's Alpha is 0.650 (Guttman's Lambda, *λ* ^2^).

^b^ Interclass Correlation Coefficient (ICC) for continuous Total Score.

^c^ Adjusted for item weighing.

^d^ Clinical complexity total.

^e^ Point‐biserial correlation coefficients for items 1–26, bootstrapped on 5000 stratified samples with tumor type as a stratification variable.

^f^ Performance status.

**TABLE 2:** Complexity levels and mean case review time durations across participating US tumor boards and overall dataset (all tumor boards), compared against the UK tumor boards (as per published data, Soukup et al., 2020).

Complexity score	US Tumor Boards				UK Tumor Boards^a^			
	≤ 1	2–3	4–6	≥ 7	≤ 1	2–3	4–6	≥ 7
Complexity level	Low	Moderate	High	Very high	Low	Moderate	High	Very high
Gynecological tumor boards (*N* = 281)								
% of case reviews	3%	8%	18%	71%	33%	34%	18%	15%
Mean review time (MM:SS)	08:26	08:47	08:58	09:36	01:28	02:11	03:13	04:38
Median review time (MM:SS)	08:57	07:06	08:21	09:05	01:15	02:00	03:00	04:00
Range review time (MM:SS)	05:47	17:11	18:52	21:08	05:09	10:59	07:35	14.10
*N* of case reviews	7	23	51	200	130	135	72	59
Urological tumor boards (*N* = 274)								
% of case reviews	0%	5%	18%	77%	—	—	—	—
Mean review time (MM:SS)	05:28	7:46	08:04	07:52	—	—	—	—
Median review time (MM:SS)	—	05:54	07:44	07:30	—	—	—	—
Range review time (MM:SS)	—	05:42	13:36	21:24	—	—	—	—
*N* of case reviews	1	14	49	210	—	—	—	—
Overall dataset (both tumor boards; *N* = 555)								
% of case reviews	1%	7%	18%	74%	27%	28%	23%	23%
Mean review time (MM:SS)	08:04	08:01	08:31	08:42	01:13	02:10	02:50	04:32
Median review time (MM:SS)	08:56	06:51	07:56	08:13	01:06	02:00	02:27	04:05
Range review time (MM:SS)	05:47	17:11	20:08	21:43	05:09	11:02	07:47	14:00
*N* of case reviews	8	37	100	410	223	228	185	186

*Note:* Case duration was not statistically different between the complexity levels, *p* > 0.05. Complexity levels are based on the quartile median values from overall dataset bootstrapped on 5000 stratified samples with tumor type as a stratification variable. Median (upper and lower bias corrected confidence intervals) for the 25th percentile was 1 (1.14–1.56), for the 50th percentile was 3 (2.99–3.46), and for the 75th percentile 6 (5.64–6.57), as reported in the original MeDiC tool study (Soukup et al., 2020).

^a^ Based on data published in Soukup et al. (2020) representing colorectal, gynecological and breast cancers.

**TABLE 3:** Reliability and Convergent Validity for the MODe Tool.

	Decision‐making factors (score range)	Assessor reliability	Item reliability	*M* (SD)		Correlation^c^			
		ICC	Cronbach's alpha if item removed^a^	Main assessor rating	2nd assessor rating	Item‐MODe Total‖		Item‐MeDiC total	
						*r*	% variability explained	*r*	% variability explained
Quality of Information (7–32; sum of items 1–7)		0.672	—	19.60 (3.728)	20.18 (3.737)	0.604*	36%	0.207*	4
1	Patient History (1–5)	0.839	0.595	4.95 (0.282)	4.96 (0.269)	0.053	0	0.017	0
2	Imaging (1–5)	0.536	0.594	4.93 (0.342)	4.97 (0.261)	0.024	0	0.098	1
3	Pathology (1–5)	0.817	0.583	4.86 (0.527)	4.89 (0.543)	0.239*	6	0.002	0
4	Psychosocial (1–5)	0.891	0.589	1.37 (1.134)	1.38 (1.156)	0.231*	5	0.217*	5
5	Comorbidity (1–5)	0.654	0.573	1.98 (1.66)	2.45 (1.896)	0.399*	16	0.044	0
6	Patient View (1–5)	0.808	0.570	1.54 (1.282)	1.80 (1.552)	0.400*	16	0.168*	3
7	Patient Seen (Y/N)‡	1.000	—	—	—	0.069	1	0.039	0
Quality of Contribution (8–37; sum of items 8 to 15)		0.815	—	20.60 (2.697)	21.43 (3.046)	0.851*	72%	0.081	1
8	Clinical Trials (Y/N)‡	0.877	—	Agree: 97v22	Disagree:2v2	−0.020	0	−0.057	0
9	Surgeon (1–5)	0.822	0.593	1.85 (0.355)	1.82 (0.385)	0.128*	2	0.020	0
10	Geneticist (1–5)	0.979	0.595	4.70 (0.975)	4.67 (0.996)	0.070	1	−0.109*	1
11	Medical Oncologist (1–5)	0.855	0.538	1.51 (0.502)	1.49 (0.502)	0.537*	29	0.217*	5
12	Radiation Oncologist (1–5)	0.587	0.569	3.64 (1.829)	4.20 (1.509)	0.417*	17	0.016	0
13	Nurse (1–5)	0.855	0.589	2.96 (1.956)	3.01 (1.918)	0.193*	4	0.076	1
14	Radiologist (1–5)	0.958	0.570	1.42 (1.145)	1.37 (1.103)	0.372*	14	−0.008	0
15	Histopathologist (1–5)	0.857	0.575	1.83 (1.535)	1.93 (1.603)	0.401*	16	−0.083	1
16	Decision made^b^	0.305	—	Agree: 112v3	Disagree:4v4	0.054	0	0.042	0
Total MODe Score (15–69; sum of items 1–15)		0.628	—	40.20 (4.82)	41.73 (5.11)	—	—	0.174*	3

*Note:* Higher scores on MODe indicate higher quality. Grayed are items that were added to the original tool. *N* of cases double‐rated =123 (22.16% of the total *N* of cases which was 555). Total *N* of meetings = 104. *N* of meetings double rated = 24 (23.08% of the total *N* of meetings).

Abbreviations: MeDiC, Measure of case‐Discussion Complexity; MODe, Metric for the Observation of Decision‐making.

* ICC, Interclass Correlation Coefficient.

^a^ Cronbach's Alpha is 0.591.

^b^ Kappa for categorical items.

^c^ Point‐biserial correlation coefficients for items 1–26, bootstrapped on 5000 stratified samples with tumor type as a stratification variable.

#### 2.7.2 Validation of MeDiC and MODe

To validate both tools, we assessed their convergent and external validity. Convergent validity was examined by computing correlations between individual factors within each tool and their respective total scores (MeDiC factors with total MeDiC score, Table 1; MeDiC‐based complexity level distributions across tumor boards, Table 2; MODe factors with total MODe score, Table 3). This analysis determined how well individual items aligned with the overall construct of complexity (MeDiC) and decision‐making quality (MODe). External validity was assessed by examining the correlations between MeDiC and MODe scores (Table 3). This analysis explored whether higher decision‐making quality, as measured by MODe, was associated with greater case complexity, as measured by MeDiC.

##### 2.7.2.1 MeDiC‐Specific Analyses

For the MeDiC tool, we examined convergent and external validity using correlations between the tool's 26 individual factors and case review duration, defined as the time from the start to the end of the patient's case discussion at the MTB. We also calculated item‐total score correlations to determine the internal consistency of individual factors within the tool. Partial correlations were computed to control for tumor type as a continuous variable, and point‐biserial correlations were used to assess associations between continuous and dichotomous factors. To further assess item validity, we calculated Item‐Content Validity Indices (I‐CVIs), frequency distributions, and associations with case review duration for each MeDiC factor. See Table 1 for these statistics.

#### 2.7.3 EMR‐Based Versus In‐Person Data Abstraction

To examine the impact of different data collection methods, MeDiC scores derived from electronic charts were compared with those obtained from direct observation. Given that chart reviews tend to provide more comprehensive clinical information, we explored whether the mode of data collection influenced complexity assessments and scoring reliability.

#### 2.7.4 Analysis of Case Complexity

Differences in case discussion time across complexity levels were analyzed using a Kruskal–Wallis H test, with complexity categories (low, moderate, high, and very high complexity) based on the classification established in Soukup et al. [14]. These complexity levels were originally derived from quartile medians in the UK‐based MeDiC validation study and were applied to the current dataset to ensure consistency in comparison. Table 2 presents the US data from this study, while the UK findings are reproduced from Soukup et al. [14] to provide contextual reference. No direct statistical comparison was conducted between the UK and US datasets. Rather, this presentation allows for an exploratory comparison of complexity distributions and discussion durations across different MDT structures. This allowed for an exploration of whether structural differences in MDT organization impact MeDiC scoring and case complexity distributions.

##### 2.7.4.1 MODe‐Specific Analyses

For the MODe tool, we examined how individual decision‐making factors contributed to the total MODe score by calculating correlations between each factor and the overall decision‐making quality measure. This allowed us to assess which factors were the strongest indicators of effective decision‐making in MTBs. We also examined how MODe scores varied with MeDiC complexity levels, assessing whether cases with higher complexity (as measured by MeDiC) corresponded to differences in decision‐making quality (as measured by MODe). See Table 3 for these statistics.

Bootstrapping with stratified sampling was employed for both MeDiC and MODe analyses, using tumor board type as the stratification variable. This approach was used to estimate confidence intervals for correlation coefficients and ensure robustness in our findings. All analyses were conducted using SPSS version 23.0, with significance set at *p* < 0.05.

## 3 Results

### 3.1 The MeDiC Tool

We assessed the MeDiC scoring and conducted psychometric analyses on 104 tumor board meetings, which reviewed 555 patients (274 urological, 281 gynecological). All 26 MeDiC items were analyzed, with their reliability coefficients, frequency counts, and correlation coefficients with the total complexity score and the case‐review duration presented in Table 1.

To evaluate how well each MeDiC item measures complexity, we used a ‘traffic‐light’ system, in line with the original UK study [14]. Green indicates an item is a strong measure of complexity (i.e., high reliability and strong correlations with total complexity). Amber represents a fair measure (i.e., moderate reliability or weaker correlations, suggesting the item is useful but may not consistently be a strong measure of complexity). Red denotes a poor measure (i.e., low reliability or weak correlations, considered for removal).

Inter‐assessor scoring reliability was evaluated using a subsample of 112 cases (20% of the total) discussed across 22 tumor board meetings. The Kappa statistics per item indicated good reliability, with coefficients ranging from 0.62 to 0.79. The ICC for the overall score was 0.849, indicating strong reliability.

Cronbach's alpha, which measured internal consistency, was marginally acceptable at 0.65, slightly lower than the originally reported value of 0.701 from Soukup et al. (2020) in the UK population. Although 94% of the overall sample was scored by the same assessors, the remaining 6% was scored by different assessors (Table S1), which could have impacted the internal consistency. However, this impact was likely minimal given the relatively small percentage of cases involved and the fact that the ICCs remained within an acceptable range. We observed a moderate concordance between MeDiC scores calculated from electronic charts versus observation data, as reflected in the ICC of 0.448, 95% CI [0.250, 0.605], *N* = 112. The MeDiC mean score was significantly higher for chart data compared to observational data: M = 5.62 for chart versus M = 4.99 for observation, *p* < 0.005, further illustrating how the mode of data collection can impact complexity assessments.

Moreover, the frequency data indicates how often each complexity factor was observed across 22 tumor board meetings analyzed. High‐frequency items suggest that these factors are commonly encountered and likely represent critical aspects of case complexity. For instance, “Malignancy”, “Invasive component”, and “Significant physical comorbidity” were among the most frequently observed factors, indicating that these are core elements of case complexity that are consistently relevant across cases. These items were also rated as “Excellent” in the Soukup et al. [14] evaluation, and their continued high performance in the current study validates their importance in assessing complexity.

On the other hand, items such as “Trial eligibility” and “Unusual anatomy/distribution of tumor” were much less frequently observed, suggesting they might represent more specialized or rare aspects of complexity, and aligning with their “Poor” or “Moderate” ratings from Soukup et al. [14]. Their low frequency and weaker correlations with total complexity suggest they might be less relevant for general complexity assessments and are potential candidates for removal or further scrutiny. In contrast to Soukup et al., where “Unusual anatomy” was rated “Excellent,” its lower frequency and correlation in the current study indicate that its utility may be more context‐dependent in US tumor boards.

The correlation data revealed how strongly each item is associated with the total MeDiC score, both unadjusted and adjusted, as well as their relationship with case duration. Items with higher correlations to the total MeDiC score indicate meaningful contributions to the overall complexity measure, while lower correlations suggest the item might not be as integral.

Items such as “Mets(metastasis) (local or distant)”, “Significant physical comorbidity”, and “Significant surgical history” exhibited strong correlations with the total MeDiC score and remain strong indicators of complexity, consistent with their evaluation in Soukup et al. [14], where they were also rated as “Excellent.” These items should be retained, as they significantly contribute to measuring case complexity.

Items such as “Malignancy” and “Increased size (T3, T4)” showed moderate correlations with the total complexity score. While these items remain still valuable, their relative contribution to complexity was slightly lower than higher‐correlation items. These factors continue to play an important role, but their performance should be monitored in future evaluations to ensure they remain reliable indicators.

Lastly, items such as “Trial eligibility” and “[Treatment] Pathway does not account for patients specific situation” exhibited low correlations with the MeDiC total score, aligning with their “Poor” ratings in both the current study and the UK validation. These factors appear less integral to the overall complexity measure and may benefit from removal or refinement in future iterations of the tool.

In addition, as shown in Table 1, the correlations with case duration were generally weaker overall, with most items showing minimal to no significant relationship with the time spent on case discussion. This finding suggests that while these items capture aspects of clinical complexity, they do not necessarily influence the length of discussions.

In contrast to previous research in the UK [14], where the four complexity levels were significantly distinct, with gradual increases in the mean time spent reviewing each patient, we found that the distribution of case discussion times was similar across all complexity levels, as assessed by visual inspection of a boxplot. The results of the Kruskal–Wallis H test were not statistically significant, *χ* ^2^(3) = 3.020, *p* = 0.389, *N* = 555, with the test statistic adjusted for ties. This pattern was consistent across both tumor boards: for the gynecological tumor board, *χ* ^2^(3) = 3.972, *p* = 0.265, *n* = 281, and for the urological tumor board, *χ* ^2^(3) = 2.656, *p* = 0.448, *n* = 274.

Summary statistics of clinical complexity across all participating MTBs, as assessed by MeDiC, are presented in Table 2, alongside UK‐based results published in Soukup et al. (2020) for illustrative comparison.

### 3.2 The MODe Tool

Table 3 provides the list of decision‐making factors within the MODe tool, along with their reliability coefficients, mean scores, and correlations with the overall MODe score as well as the MeDiC score.

Inter‐assessor reliability was assessed using a subsample of 123 cases (22% of the total) discussed across 24 meetings. Intraclass correlation coefficients (ICC) per item indicated moderate to strong reliability, ranging from acceptable to high agreement depending on the item. The overall ICC for the MODe tool was 0.628, which demonstrates moderate reliability for evaluating decision‐making factors, which is in line with previous findings but slightly lower than expected in some areas [16, 17].

Cronbach's alpha, which measures internal consistency, was marginally acceptable at 0.591. While previous research on the MODe tool [17] reported Cronbach's alpha of 0.715, the lower internal consistency in this study may be attributed to variability in the second assessors. Although the first assessor was consistent across all cases and all assessors were calibrated and trained, the second assessors varied across cases, and in some instances, the first assessor participated virtually while the second was physically present.

The “Quality of Information” and “Quality of Contribution” factors both showed acceptable to strong correlations with the overall MODe score. Key items, such as “Patient View”, “Comorbidity”, and “Medical Oncologist Contributions”, exhibited significant correlations with the total MODe score, indicating that these factors are strong contributors to decision‐making quality. In particular, “Comorbidity” and “Medical Oncologist” contributions were highly influential in defining decision‐making quality, reflecting their central role in complex case discussions.

Items such as “Pathology” and “Radiation Oncologist” contributions also showed moderate correlations with the overall MODe score, suggesting that they are meaningful components of the tool but not as critical as the higher‐correlating items. These items should continue to be monitored in future evaluations to ensure they maintain their relevance in assessing decision‐making quality.

A few items, such as “Imaging” and “Trial Eligibility”, exhibited weaker correlations with the overall score and were less frequently observed across the sample. These items may represent specialized factors that do not contribute as consistently to decision‐making quality across gyn and uro cases; however, they may have a higher impact in other specialties, esp. imaging, in regard to complex surgical resections. They may warrant further review in future iterations of the tool to determine if they should be retained or modified.

## 4 Discussion

To the best of our knowledge, this study offers the first validation of the MeDiC tool [14] in U.S. tumor boards to assess the clinical complexity of cancer patients managed in this setting, as well as the MODe tool [16] to evaluate the team decision‐making process. Our findings revealed that over 70% of the cases discussed were classified as a very high complexity, with 74% of the total across both tumor boards falling into this category, and an additional 18% classified as high complexity. This distribution likely reflects the study's setting at a tertiary care center, where tumor boards primarily manage highly complex cases, as opposed to community hospitals, which may handle a broader spectrum of cases, including lower‐complexity discussions [5]. Given that tertiary centers often serve as referral hubs for complex, advanced, or recurrent cases, the resulting case mix may limit the direct generalizability of these findings to community or comprehensive cancer centers, where low‐ and moderate‐complexity cases are more frequently discussed, and where MeDiC score distributions and their relationship to decision‐making processes may differ. Future research should therefore validate MeDiC and MODe across a wider range of institutional contexts, including multi‐site studies deliberately sampling tumor boards with heterogeneous case mixes, to assess whether the tools perform consistently across settings and whether context‐specific implementation strategies are required.

The analysis also confirmed the validity statistics for both tools and provided key insights into their optimal use, particularly in identifying areas for process improvement and standardizing tumor board evaluations as part of a toolkit for tumor board assessment and improvement. The validation of MeDiC and MODe in the US context provides a practical foundation for integrating these tools into tumor board workflows to support process optimization, guide targeted staff training, and inform institutional or national policies on MDT function and accountability. Future research should explore the applicability of these tools in community hospital settings, where case complexity, workflow dynamics, and decision‐making structures may differ.

### 4.1 Lessons Learned and Recommendations for Future Use of MODe and MeDiC

#### 4.1.1 Feasibility and Documentation Practices

Our experience with the MeDiC tool highlighted the importance of using chart reviews, provided that the tumor board consistently documents their meetings in a formal “tumor board note.” This refers to the structured documentation of tumor board discussions and recommendations after the MDT, rather than a patient summary prepared in advance. While tumor board notes are not designed for pre‐meeting preparation, their consistent use enhances retrospective evaluations and ensures reliable data collection, which is particularly valuable for quality improvement and validation studies. The absence of standardized tumor board documentation presents a challenge in some settings, as inconsistencies in record‐keeping can limit the reliability of complexity assessments and decision‐making evaluations [12, 22, 23]. Given the critical role of tumor board discussions in guiding patient care, the implementation of standardized documentation practices could support both clinical decision‐making and future research evaluating tumor board effectiveness. This was evident in the gynecological oncology tumor board which had consistent documentation unlike the urology tumor board. The absence of such documentation in urology led to variability in the information available, depending on the primary provider. For example, while some providers offered detailed medical histories and diagnoses, others often had sparse or missing documentation. This suggests that using the MeDiC tool consistently—whether through chart reviews or real‐time observations—is crucial for achieving consistency and reliable results. Standardized use of structured tumor board notes, as promoted in recent guidelines [22], could further enhance the tool's reliability by ensuring uniform data availability and reducing variability in complexity assessments.

#### 4.1.2 Standardization and Methodology in MeDiC Scoring

Maintaining consistency in data collection methods is crucial for ensuring reliable MeDiC scoring. While both chart‐based and observation‐based assessments can be effective, differences between these methods highlight the importance of standardized documentation practices in tumor boards. Where feasible, structured tumor board notes could improve the accuracy and reproducibility of complexity assessments, particularly for teams relying on retrospective data [15].

Originally, MeDiC was tested in real time during tumor board meetings [14], however, its intended use is to help with meeting preparation, streamlining the review process, and prioritizing cases based on complexity. For instance, generating a complexity score when a case is first selected for MTB review could help in organizing cases on the agenda, starting with the most complex. Consistency in assessors and data collection methods, whether through charts or observations (one or the other), is key to maintaining the tool's reliability and reproducibility.

#### 4.1.3 Validation and Interpretation of Complexity Assessments

As shown in Table 2, more than 70% of urological and gynecological cases fell within the top 25% in terms of complexity, indicating a concentration of very high complexity cases. This suggests that case duration may not be an entirely accurate external validation metric for some tumor boards [24]. Specifically, in health systems such as the ones in the UK, where all cases are discussed at tumor boards, there is greater variability in case complexity, making case duration a valid metric. However, in health systems such as in the US, where tumor boards primarily focus on highly complex cases, the lack of simpler cases results in reduced variability in complexity levels. This homogeneity limits the usefulness of case discussion duration as an external validation metric, as duration represents a relatively limited proxy for clinical complexity, particularly in settings where tumor boards predominantly review highly complex cases. In such contexts, restricted variability in case mix reduces the ability of discussion time to discriminate meaningfully between different levels of complexity. Future research could strengthen the external validity of MeDiC and MODe by examining their associations with complementary process‐ and outcome‐oriented indicators, such as adherence to guideline‐concordant care, timeliness of treatment initiation, or downstream care coordination outcomes. Importantly, these measures should be interpreted within the intended scope of the tools: MeDiC is designed to characterize case complexity and support prioritization and workflow decisions, while MODe evaluates the quality of multidisciplinary decision‐making processes rather than clinical outcomes per se. Linking these tools to downstream indicators will therefore require carefully designed studies that account for contextual factors, case selection practices, and the non‐linear relationship between decision‐making quality, complexity, and patient outcomes.

An interesting finding was that factors such as malignancy and tumor size (T3, T4) showed only moderate correlations with overall complexity. Given that higher‐stage disease is a key driver of treatment decisions, particularly in the US, this result may reflect differences in how complexity is perceived and evaluated across healthcare settings. In US tumor boards, early‐stage (T1) cancers are less frequently presented, leading to a case mix skewed toward more advanced disease. As a result, tumor size may not emerge as a dominant factor in complexity scoring, since most cases already involve higher‐stage disease, reducing its relative discriminatory power. Future research could examine how differences in case selection influence complexity assessments. If tumor boards primarily review high‐stage cancers, certain factors, such as tumor size, may contribute less to overall complexity simply because they are common across cases. Conversely, in settings where a broader range of cases is discussed, these factors may hold greater predictive value. Understanding these dynamics could help refine how complexity is measured and interpreted across different healthcare systems.

#### 4.1.4 Internal Consistency and Reliability of MODe and MeDiC

The internal consistency estimates observed for both MeDiC and MODe should be interpreted in light of the tools' structure, purpose, and real‐world application. Both instruments are intentionally multidimensional, capturing heterogeneous but clinically relevant components of case complexity (MeDiC) and decision‐making quality (MODe), rather than reflecting a single latent construct. In this context, Cronbach's alpha values modestly below conventional thresholds do not necessarily indicate poor reliability but rather reflect the diversity of contributing domains assessed within each tool. Variability in second assessors and differences in data collection methods further highlight the importance of standardization in routine practice. Consistency may be improved through the use of a single, clearly defined mode of data abstraction (e.g., structured chart review or real‐time observation), consistent pairing of assessors where feasible, and formalized refresher training to maintain calibration over time. In addition, embedding the tools within standardized tumor board documentation practices—such as structured tumor board notes—could reduce information variability and support more stable scoring across assessors and settings. Together, these considerations highlight that reliability is influenced not only by the psychometric properties of the tools themselves, but also by how they are implemented within clinical workflows [16, 17, 20].

A key factor influencing the internal consistency of the MeDiC tool was inconsistency in data collection methods, with some cases relying on direct observations and others scored using medical records or chart reviews. Charts typically provided more comprehensive clinical information, resulting in higher scores, while observation‐based assessments may have missed key details due to reliance on verbal communication during meetings. Additionally, qualitative reflections from assessors indicated that chart‐based assessments offered greater completeness and confidence in scoring. These experiences suggest that the perceived superiority of chart review may reflect both the depth of information and the level of assessor familiarity with the tool and context. This approach is also more closely aligned with the intended use of the MeDiC tool, which was designed to be scored based on information available in clinical records or charts [14]. While observation‐based scoring was used during the tool's initial development phase, this was primarily for construct development and feasibility testing, not as a recommended mode of application in routine or evaluative use.

For the MODe tool, variability in second assessors and assessment environments may have contributed to minor fluctuations in internal consistency compared with previously reported values [16, 17, 20]. Ensuring consistency in assessor pairing and aligning virtual and in‐person scoring conditions in future applications may help further enhance reproducibility across tumor board settings [17].

The performance of certain MODe factors varied across tumor board settings, with some items showing weaker correlations with overall decision‐making quality. For example, Imaging and Trial Eligibility contributed less consistently to decision‐making in gynecological and urological tumor boards, despite their potential relevance in other specialties. This may reflect differences in how certain disciplines integrate imaging data into decision‐making or how frequently clinical trial options are considered within these specific MDTs. Taken together, these findings suggest that items such as Imaging and Trial Eligibility may reflect context‐ and specialty‐specific dimensions of complexity or multidisciplinary decision‐making, rather than deficiencies in the tools themselves. At this stage, these findings do not warrant item removal. Instead, they support a cautious, evidence‐informed approach to future refinement. Planned future work will examine item performance across a broader range of tumor boards, cancer types, and healthcare settings to determine whether item weighting, contextual tailoring, or specialty‐specific adaptations are more appropriate than universal item modification.

These findings suggest that the relative importance of MODe factors may be influenced by tumor board composition and case mix. For instance, in surgical oncology MDTs for pancreatic or liver cancer, imaging is central to determining resectability, guiding surgical margins, and evaluating response to neoadjuvant therapy [20]. In contrast, for tumor types where systemic therapy dominates, such as hematologic cancers or metastatic melanoma, clinical trial eligibility may be a critical factor influencing treatment decisions, particularly in the context of novel immunotherapies. Future studies could explore whether these factors hold greater weight in MDTs managing more complex surgical resections or specialties where imaging plays a more central role. Refining MODe for different tumor board contexts could enhance its ability to capture specialty‐specific decision‐making dynamics.

The strong influence of “Comorbidity” and “Medical Oncologist Contributions” in defining decision‐making quality aligns with their central role in complex cancer cases. Comorbidities often shape treatment feasibility, toxicity risk, and overall prognosis, making them a crucial factor in selecting appropriate therapeutic strategies, particularly for patients with multiple chronic conditions. Given that many cancer treatments require balancing efficacy with tolerability, the presence of comorbidities frequently necessitates adjustments in treatment plans, multidisciplinary input, and risk–benefit discussions. Similarly, medical oncologists play a pivotal role in MDT decision‐making as they typically lead discussions on systemic therapy options, including chemotherapy, immunotherapy, and targeted treatments. Their contributions directly influence treatment sequencing, suitability for clinical trials, and the integration of emerging therapies. This may explain why medical oncologist input was consistently weighted more heavily in defining decision‐making quality. These findings highlight the importance of ensuring strong interdisciplinary engagement in MDTs, particularly when dealing with complex, multimodal cancer care decisions.

### 4.2 MeDiC and MODe as Part of the Tumor Board Evaluation Toolkit

Both the MeDiC and MODe tools can significantly enhance the structure and efficiency of MTBs. In health systems like the US, where only selected cancer patients are reviewed, MeDiC could help standardize case selection based on complexity, prioritizing complex cases for full review and managing simpler ones through established guidelines, reducing MTB workload [14, 15]. In the UK, where tumor boards review all cancer cases, MeDiC could address concerns over sustainability by enabling complexity‐based screening to ensure more in‐depth reviews of complex cases, while simpler cases follow predefined protocols [25]. In health systems without MTBs, MeDiC could support phased implementation by selecting patients based on complexity, preventing resource overload and facilitating gradual integration of MTB practices.

Given the variability in case complexity across tumor types and functioning of individual tumor boards, one MTB implementation design is unlikely to fit all contexts. For example, MTBs at tertiary cancer centers handle the most complex cases, requiring tailored processes to manage caseloads effectively—the MeDiC tool could enable these centers to redesign care processes while maintaining adequate governance. Meanwhile, the MODe tool could help identify areas within tumor boards that either promote or hinder holistic patient reviews, offering a framework for continuous quality improvement.

Specifically, the MODe tool allows for the tumor boards to identify areas that promote or hinder holistic patient review within a meeting and make recommendations that are both clinically sound and acceptable to patients. It also allows self‐reflection among team members based on the data and identification of appropriate strategies/interventions for improvement that are team driven. One way of initiating such improvement is to apply the principles of “team audit and feedback”—a process of providing non‐punitive and actionable feedback to professionals to allow them to self‐assess and adjust their performance, thus stimulating desired behavior change [18]. Such an approach was found effective in improving practice and supporting quality improvements, and it can be used to aid implementation of evidence‐based interventions [18]. In previous work [18], this approach allowed eliciting inputs from all team members, which were then used to co‐design interventions to best meet the needs of the team.

### 4.3 Contextualizing the UK Versus US Comparison

While UK and US tumor boards share the goal of multidisciplinary collaboration, they differ structurally and operationally. UK MDTs typically review all newly diagnosed cancer cases, whereas US tumor boards selectively focus on complex cases due to resource constraints. This fundamental difference affects workflow, team engagement, and the nature of decision‐making processes. Therefore, in this study, UK versus US differences are examined not as a central comparison, but within the context of psychometric validation, to understand how MeDiC and MODe function in different healthcare systems and how these differences may impact quality of decision‐making and MTB efficiency.

### 4.4 Limitations

The use of different assessors may have influenced internal consistency, with greater variability stemming from differences in the mode of data collection as some cases relied on direct observations while others used medical records or chart reviews. Charts typically provide more comprehensive clinical information which can be reviewed at the assessor's convenience and pace, resulting in higher scores, while observation‐based assessments may have missed key details due to reliance on verbal communication during meetings.

One limitation of this study is that the case mix at our study site may not be representative of all US tumor boards. The concentration of very high‐complexity cases observed in this study may reflect institutional factors, such as the tumor board's role within a tertiary referral center or specific patient referral pathways. While the findings provide valuable insight into complexity distribution within US tumor boards, case mix variability across institutions—particularly between academic centers, community hospitals, and comprehensive cancer centers—should be considered when interpreting these results. Future studies could examine whether complexity distributions differ across diverse healthcare settings to better understand how institutional factors shape MDT decision‐making.

## 5 Conclusion

Both MeDiC and MODe offer evidence‐based tools that allow cancer teams to streamline patient selection and decision‐making processes while providing a means for self‐assessment and quality improvement. The implementation of these tools and the study of their impact on patient case selection, workflow efficiency, and cost of care will help policymakers optimize MTB‐driven care. Additionally, MODe can serve as a tool for comparing decision‐making processes across different MTBs, supporting efforts to standardize multidisciplinary discussions and improve consistency in clinical decision‐making. This, in turn, could enhance the quality and efficiency of cancer care delivery across various health systems.

## Author Contributions

**T. Soukup:** conceptualization, investigation, funding acquisition, writing – original draft, methodology, validation, writing – review and editing, formal analysis. **B. W. Lamb:** writing – original draft, methodology, validation, writing – review and editing. **K. Noyes:** investigation, funding acquisition, writing – original draft, writing – review and editing, methodology, project administration, supervision, conceptualization. **N. Lajmi:** conceptualization, funding acquisition, writing – original draft, writing – review and editing, resources, project administration, software. **W. Zhang:** investigation, writing – original draft, writing – review and editing, data curation, project administration. **K. Stuart:** investigation, writing – original draft, writing – review and editing, project administration, data curation. **G. Markollari:** investigation, writing – original draft, writing – review and editing, project administration, data curation. **M. Wallace:** investigation, writing – original draft, writing – review and editing, project administration, supervision. **S. Perrigo:** investigation, writing – original draft, writing – review and editing, project administration, data curation. **M. S. Prime:** conceptualization, funding acquisition, writing – original draft, writing – review and editing, project administration, resources, software. **R. Ortiz:** investigation, writing – original draft, writing – review and editing, project administration, data curation. **R. D. Hammer:** conceptualization, investigation, funding acquisition, writing – original draft, methodology, validation, writing – review and editing, project administration, supervision, resources.

## Funding

This project was funded by Roche Molecular Systems Inc. Pleasanton, CA, US.

## Conflicts of Interest

Richard D. Hammer MD participates in consulting, speaking, and sponsored research with Roche, Basel Switzerland, and unfunded collaborative research with Foundation Medicine, Cambridge MA. Matthew S. Prime and Nesrine Lajmi are employed by Roche. In line with the guidelines by the International Committee of Medical Journal Editors, all authors for this study have made substantial contributions to conception and design, or acquisition of data, or analysis and interpretation of data; have been involved in drafting the manuscript or revising it critically for important intellectual content; have given final approval of the version to be published; and have agreed to be accountable for all aspects of the work in ensuring that questions related to the accuracy or integrity of any part of the work are appropriately investigated and resolved.

## Supporting information


**Table S1:** Frequency and percentage of meetings assessed per assessor.

## Data Availability

The data that support the findings of this study are available on request from the corresponding author. The data are not publicly available due to privacy or ethical restrictions.
