# Supplementary material for: Validation of the Measure of Case‐Discussion Complexity and the Metric for the Observation of Decision‐Making for Streamlining Workflow and Evaluating Decision‐Making in US Tumor Boards
Source: Cancer Med. 2026 May 29;15(6):e71970. doi: 10.1002/cam4.71970 (PMC13240311; doi:10.1002/cam4.71970)
Supplement: Supplementary file 1 — Table S1: Frequency and percentage of meetings assessed per assessor. [file CAM4-15-e71970-s001.docx]

**Supplementary Table 1.** Frequency and percentage of meetings assessed per assessor

|  | **Subsample for inter-assessor reliability analysis*** | | | | **Entire sample**** | | |
| --- | --- | --- | --- | --- | --- | --- | --- |
| **Assessor** | MODe Main Assessor | MODe 2^nd^ Assessor | MeDiC Main Assessor | MeDiC 2^nd^ Assessor | MODe  Assessor | MeDiC Assessor | **Total**  ***N*** |
| **1=SP** | 23 (96%) | - | - | - | **99 (94%)** | 1 (1%) | 100 |
| **2=MW** | - | - | **6 (27%)** | **3 (14%)** | **-** | **36 (35%)** | 36 |
| 3=L | - | 1 (4%) | - | 2 (9%) | - | 1 (1%) | 1 |
| **4=KS** | 1 (4%) | 14 (58%) | - | - | 1 (1%) | - | 1 |
| 5=WZ | - | 5 (21%) | - | 10 (45%) | 1 (1%) | 5 (5%) | 6 |
| 6=RO | - | 4 (17%) | - | 7 (32%) | 3 (3%) | 3 (3%) | 6 |
| **7=GM** | - | - | **16 (73%)** | **-** | **-** | **58 (56%)** | 58 |
| **Total *N*** | 24 | 24 | 22 | 22 | 104 | 104 | - |

*Note.* A subsample of 112 cases and 22 meetings double-assessed using MeDiC, and 123 cases and 24 meetings double-assessed using MODe (~20% of total sample), for inter-assessor reliability analysis. **Entire sample of 555 cases and 104 meetings.
